# Exploring the trend in religious diversity: Based on the geographical perspective

**DOI:** 10.1371/journal.pone.0271343

**Published:** 2022-07-14

**Authors:** Xiaobiao Lin, Qinghe Chen, Luyao Wei, Yuqi Lu, Yu Chen, Zhichao He

**Affiliations:** 1 School of Geographical Science, Nanjing Normal University, Nanjing, Jiangsu, China; 2 School of Geography and Tourism, Anhui Normal University, Wuhu, Anhui, China; 3 Department of Sustainable Landscape Development, Institute for Geosciences and Geography, Martin-Luther-University Halle-Wittenberg, Halle (Saale), Germany; Sun Yat-sen University, CHINA

## Abstract

The formation and development of religious diversity is a manifestation of the free expression of human thought, belief, and practice, as well as a historical premise and ideological condition for the gradual recognition and integration of modern religions into modern political values. This study examines the spatial characteristics of the development of the global religious diversity index (RDI) and the evolution trend through a geographical perspective by the LISA space-time transition and convergence test. The results show that: (1) At the temporal level, RDI showed a fast and then slow increase after WWII, with an increase of 61.11%. (2) At the spatial level, Latin America has seen the most significant increase in RDI, followed by Europe, North America and the Caribbean, while Asia has a slight decrease. (3) At the country level, most countries with the highest levels of RDI are located in North America and the Caribbean, Sub-Saharan Africa, and most of these countries have a history of being colonized. RDI was mainly influenced by factors such as the missionary effect in the colonial period, precipitation, GDP per capita, and genetic diversity. (4) The evolution of the spatial structure of global RDI has a certain path-dependent, but this trend is gradually weakening. In addition to countries’ own development, RDI is also influenced by spillover effects from the neighboring countries. (5) There is a significant σ convergence and absolute β convergence in the global RDI, and most of the continental units have club convergence, i.e., the internal differences in RDI levels at the global and regional levels are gradually narrowing, and there is a spillover effect of higher RDI levels to the surrounding lower regions, and this diffusion or influence allows the lower regions to catch up in the gap of RDI.

## Introduction

Religious diversity is a social phenomenon in which two or more clearly defined religions exist simultaneously within a region or society. Pluralism believes that no one religion has a unique approach to religious truth and that all religions can and should respect, tolerate, and peacefully coexist with each other [1, 2]. Due to the wave of global migration, the construction of transportation infrastructure, and the spread of Internet communication technology, religions have been introduced to areas that previously had a single religion, and the speed of spread and the degree of communication between religions is far greater than any other time in history [3]. Peter Berger points out that with modernization and globalization, religion is not in decline, but is experiencing a more diverse development [4]. Global integration has made the interconnection of religions closer, and interreligious ties and interactions, such as conflicts, communication, competition, and cooperation, have a growing impact on the world. This has prompted religious researchers to broaden their horizons and adopt spatial thinking to examine and reconstruct the intricate network of relationships, spatial distribution, and comparative influence among religions, focusing on their evolutionary dynamics and spatial competition mechanisms. This geographical way of thinking enables a more systematic and in-depth understanding of the ways and mechanisms by which religion exerts its influence on global politics, economy, culture, and other areas in the context of globalization.

Early research on religious diversity focused on the link to religious beliefs—that is, the extent to which religious diversity affects believers’ participation in religious activities. Traditional secularization theory suggests that religious diversity reduces religious participation and that individuals who are exposed to multiple religions will weaken their devotion to a particular religion [5]. Finke et al. offer a different perspective on this issue, arguing that religious diversity stimulates interfaith competition and provides individuals with more choices, leading to greater religious participation and higher church attendance [6]. As the study of religious diversity deepens, its impact on economic activities is gradually becoming a new research focus, and some scholars have found that religious diversity can promote religious mobilization and increase the vigor of religious economies [7, 8]. Barro et al. expanded on this research to demonstrate that countries with higher religious diversity have better economic growth prospects [9]. Following a similar methodological path, Helble and Dolansky explore the impact of religious diversity on investment, showing that countries with greater religious diversity tend to be more active in investment and economic cooperation. They explain that countries with higher religious diversity tend to be more flexible, open, and more willing to face risks. At the individual level, people in more religiously diverse environments are generally more tolerant and performant and therefore more inclined to communicate, interact and cooperate. Helble similarly finds a positive effect of religious diversity on economic activity over religious similarity, arguing that while shared religious beliefs may promote cross-country investment and trade, the endogenous dynamics of investment and trade between different religious faiths tend to be stronger due to heterogeneous preferences for product demand across religions [10, 11]. Mina obtained similar findings when she studied the impact of religion on FDI in middle-income countries [12]. Hergueux et al. further deepen the study and find that the economic impact of religious diversity varies significantly, often mediated by the political system in international economic and commercial cooperation. They argue that the impact of religious diversity on different types of countries depends mainly on the efficiency of government institutions and that high religious diversity may lead to greater inter-communal tensions in countries with weak state systems, while in countries with better political systems it can have a positive impact on economic development and investment building [13]. Other studies of religious diversity have covered areas such as political economy [14–16], and biomedicine [17].

In contrast, the study of religious diversity in geography has developed later but is more focused on scale and geographical differentiation. For example, Warf has analyzed the spatial distribution of religious diversity with global, North American, and U.S. studies and summarized the driving factors including national history, population growth rate, migration trends, etc [18–20]. Torre et al. map religious distribution with the latest data on religious affiliation in Mexico and identify key factors and trends in this transformation as a way to reflect on local religious transformation issues and challenges [21]. Mapping and measuring religious diversity has arguably become the key to managing interreligious relations in the 21st century [22]. In addition, Ying et al. examined the potential impact of religious diversity on regional development in China using data on religious sites, demonstrating that religious diversity has significant spatial differences within regions [23].

In general, the current research on religious diversity is mainly based on socio-economic theories, studying its effects and transmission mechanisms as a cultural element or social capital in various aspects. There is a lack of quantitative research on religious diversity as an object of study. In terms of research methods, previous studies have focused on using econometric theory to determine the computational model of religious diversity, and on this basis to examine its external influence through traditional methods such as linear regression, and many new techniques and methods in geography have not been applied and tested. In terms of research stages, almost all existing studies focus on the distribution of religious diversity in cross-sectional states and lack macro-scale studies of long time series. Moreover, from the perspective of development, there are bound to be significant regional differences in the religious diversity in different regions of the world due to their respective social development status and cultural underpinnings. So will such differences gradually decrease and converge over globalization? It is clear that this issue is important for understanding religious phenomena and solving global religious problems in the context of globalization.

Therefore, this paper attempts to explore the spatial distribution characteristics and pattern evolution of global religious diversity in the post-World War II period more systematically and comprehensively from the perspective of religious geography, using spatial and economic geography methods such as LISA space-time transition and convergence tests, and to demonstrate its convergence in the development process and its influencing factors on this basis. We aim to expand the perspective of religious geography research and provide the theoretical and practical basis and decision-making reference for religious issues in the context of globalization.

## Material and methods

### Research objects

This paper focuses on a total of 203 countries/regions worldwide during the 70 years from the end of World War II to 2015 (including countries such as the Soviet Union, Yugoslavia, Czechoslovakia, South Vietnam, East Germany, and South Yemen that survived and dissolved during the study period. In addition, data on colonies and overseas territories are not included in the homeland). The research phase was divided into 15 periods using the end of 1945 as the base period and 5 years as one period. For the continental division, taking into account the regional differences in cultural geography and referring to the United Nations database (UNDATA) criteria, the global countries are divided into seven continental units based on the relative cultural homogeneity of the regions: Europe, Asia (except West Asia, the same below), West Asia and North Africa, Sub-Saharan Africa, North America and the Caribbean, Latin America, and Oceania.

### Religious division

For the purpose of research data statistics, comparison, and analysis, reference Religion Family Trees classifies global religions according to denominations and sects and group them together bottom-up, such as classifying Lutheran, Calvinist, and Baptist into the scope of Protestantism, Sunnis and Shiites into Islam, and Mahayana, Theravada into Buddhism. Although according to a 2017 Pew Research Center survey, the Hindu population (1.10 billion) was already twice as large as the Buddhist population (0.49 billion) in 2015 and will further widen the gap in the future. Based on the audience of believers, the scope of dissemination, and the global influence, we divide world religions into seven categories: Protestantism, Catholicism, Orthodoxy, Islam, Buddhism, Other religions, and non-religion which also fit with the traditional definition of the three major world religions division.

### Data sources

Post-World War II global population data by religious affiliation were obtained from the Association of Religion Data Archives (ARDA) RCS-Dem dataset and The World Religion Project (WRP), co-sponsored by the University of California, Davis and Pennsylvania State University [24, 25]. The data includes religious population panel statistics for more than 200 countries/regions worldwide for the last 200 years and estimates the population of adherents of more than 100 religious denominations worldwide by year and their population proportions. We collated, extracted, and grouped the data according to the previous religious classification guidelines as the primary data source for this study.

In addition, GDP per capita data were obtained from the World Bank, agricultural suitability of land, and precipitation data from the FAO, comprehensive national power data from the National Material Capabilities Program (NMC) hosted by Pennsylvania State University, and genetic diversity and colonization data from existing studies by Ashraf and Ziltener [26–28].

### Research methods

#### Calculation of religious diversity index (RDI)

Few existing studies on religious diversity contain explicit methodological statements, probably because the development of a rigorous methodology requires the immediate confrontation of two difficult issues. First, how do we define the abstract concept of diversity? Second, how can diversity be defined in terms of an integrated understanding that makes sense for religion? A precise definition of diversity is a prerequisite and basis for robust quantitative comparisons, so the clear division of religion above not only distinguishes diversity from simple richness but also captures differences between levels or kinds of diversity [29, 30]. Due to the difference in the understanding of diversity, it can have different meanings depending on the measurement method, which can lead to differences in the calculation results. For example, the Harvard Pluralism Project Shows that "the United States has become the most religiously diverse nation on earth " [31]. By contrast, the Pew study concludes that, as of 2014, the United States ranks only sixty-eighth of 232 countries and territories assessed in terms of their religious diversity [32].

Methodologically, The differentiation index is often used to characterize religious diversity because of its simplicity, and ease of use [33, 34]. However, in the face of the richness and complexity of social science concepts, it is clear that this approach cannot fully cover and represent religious diversity [35]. Another measurement of religious diversity is the index of religious polarization proposed by Montalvo and Reynal-Querol, who argue that religious polarization might well capture the extent of religious conflict better than religious differentiation [36]. Although the polarization index takes into account religious competition and to some extent improves on the religious polarization index, there are still problems that the definition is too narrow and not all religions are in competition with each other [37]. Considering that the differentiation index and the polarization index are significantly correlated and have complementary meanings, the most robust strategy for measuring religious diversity is a multi-year, multi-method measure of the evaluation unit after integrating the two methods [38]. By combining the differentiation Index and polarization index, we could be more confident about both the substantive meaning and arithmetic comprehensiveness of contextual religious diversity. Hereby, we address the two difficult issues by dividing religion into Christianity, Catholicism, Islam, Buddhism, other religions, no religion (to prevent small religions from being overweighted), and equalizing the index of differentiation and polarization. That is, the “diversity” in this study emphasizes the relative balance and stability of religious forces within a region or country while expressing the richness of religion. At the same time, this setting also has social significance as opposed to simply considering religious richness or concentration [13]. The specific method is accounted for by the Herfindahl-Hirschman index and the polarization index.

As a common derivative form of the differentiation index, the Herfindahl-Hirschman index was originally used by economists to measure product market concentration and has been used in many cultural studies in recent years. It characterizes the degree of dispersion of the data, i.e., the influence of multiple religions within a cluster, by calculating the probability of subsamples belonging to different religions under the system, taking values between 0 and 1. When the value is 1, all individuals belong to different religions. conversely, when the value is 0, all individuals belong to the same religion. The polarization index is another measure of religious diversity, which combines a dispersed sample into a cluster according to criteria. A cluster is considered polarized if the attributes of samples within the same cluster are homogeneous, while samples between different clusters have different attributes. Based on this theory, various measures of religious diversity have been developed, among which the Montalvo polarization index is the most widely used [39]. The two indices have their emphasis point in terms of indicator meaning, such as the Herfindahl-Hirschman index, also known as the index of differentiation, which describes the impact of multiple religions within a country/region, while polarization describes the impact of the degree of differentiation between religions within a country/region. The integrated calculation formula is:

RDI=H+P2H=1-∑i=1Nπi2P=1-∑i=1N(12-πi12)2πi (1)

Where: RDI is the religious diversity index; H is the religious differentiation index; P is the religious polarization index; π_*i*_ is the proportion of the population of religion i to the population of all religions; N is the number of religion.

#### LISA space-time transition

LISA (local indicators of spatial association) is often used to reveal the spatially dependent characteristics of geographical elements. As its derivative, LISA space-time transition can represent the temporal variation of neighborhood spatial patterns (HH, LH, LL, HL, who are distributed in the first to fourth quadrants in that order) on Moran’s I scatter diagram, as a way to dynamically analyze the evolution pattern of the study object at the spatio-temporal level. Specifically, HH or LL means that a country has a high or low level of RDI in both itself and its neighbors. HL, LH indicates that two neighboring countries and regions belong to different levels. By embedding the spatial attributes such as distance and direction of the geographic coordinates of the study area in Moran’s I scatter diagram into the traditional Markov chain, local Markov transition and spatio-temporal transition are proposed and classified into four transition types (Type 0, TypeI, TypeII, and Type III) [40]. Specifically, Type 0 indicates that no morphological transition has occurred in the study area over time, and all lie on the main diagonal of the transfer matrix. Type I indicates that the transition itself occurs while the neighboring area remains unchanged, including the following possibilities: HH_t_ → LH_t+1_, HL_t_ → LL_t+1_, LH_t_ → HH_t+1_, LL_t_ → HL_t+1_ (t is the starting year). Type II indicates that it remains unchanged while the neighboring areas are transitioned, including the following possibilities: HH_t_ → HL_t+1_, HL_t_ → HH_t+1_, LH_t_ → LL_t+1_, LL_t_ → LH_t+1_. Type III indicates that both self and neighboring areas have transitioned. It can be further subdivided according to the transition direction, if the transition directions are the same, it is Type IIIA, including HH_t_ → LL_t+1_, LL_t_ → HH_t+1_. If the transition direction is opposite, it is Type IIIB, including HL_t_ → LH_t+1_, LH_t_ → HL_t+1_. On this basis, spatio-temporal flow (SF, indicates the degree of elemental flow in a certain time and space range) and spatio-temporal cohesion (SC, indicates the degree of elemental solidification in a certain time and space range) are set as the ratio of the number of a certain leap type to its possible number of all leaps in a certain time frame [41].

#### Convergence test

On the basis of the LISA space-time transition reflecting the spatial correlation and dynamic evolution of the global religious diversity, the convergence model is introduced for further analysis to accurately portray the spatial heterogeneity trend of the global and local regions. Convergence of RDI means that the regional disparity of religious diversity tends to decrease over time. Convergence methods mainly include σ convergence, absolute β convergence, and club convergence.

σ convergence. σ convergence refers to the decreasing trend of regional differences over time, expressed as coefficient of variation, i.e., the gradual decrease in the coefficient of variation of regional RDI represents its existence σ convergence. The formula is as follows:

$$\text{CV}=\sqrt{\frac{1}{\text{n}}\sum_{\text{i}=1}^{\text{n}}{\left({\text{RDI}}_{\text{i}}\text{-}\frac{1}{\text{n}}\sum_{\text{i}=1}^{\text{n}}{\text{RDI}}_{\text{i}}\right)}^{2}}/\left(\frac{1}{\text{n}}\sum_{\text{i}=1}^{\text{n}}{\text{RDI}}_{\text{i}}\right)$$ (2)

Where: CV is the coefficient of variation; RDI_i_ is the religious diversity index of country/region i; n is the number of countries/regions.

β convergence. β convergence is usually divided into absolute and conditional β convergence. The absolute convergence assumes that the conditions for the development of religious diversity are identical across regions and converge to the same steady-state level. The formula is as follows:

$$\text{In}\;{\text{(RDI}}_{\text{i},\;\text{t}+\text{T}}/{\text{RDI}}_{\text{i},\;0}\text{)}=\alpha +\beta \text{In}\left({\text{RDI}}_{\text{i},\;\text{t}}\right)+{\text{h}}_{\text{i}}+{\text{k}}_{\text{t}}+\varepsilon_{\text{i},\;\text{t}}$$ (3)

Where: In (RDI_i, t+T_/RDI_i, t_) denotes the growth rate of country/region i’s RDI from year t to t+T; RDI_i, 0_ is the RDI of the ith country/region at the beginning of the study period; RDI_i, t_ is the RDI of the i-th country/region in period “t”; α, β are parameters to be estimated; T is the time span; h_i_, k_t_ are the individual effects that do not vary over time and the time effects that do not vary over time, respectively; *ε*_*i*,*t*_ is the random error term. Convergence speed θ = -ln(β+1) / T, if β is less than 0 and significant, then conditional convergence is considered to exist.

Club convergence. For the consideration of exploring the spatial heterogeneity within the region, club convergence is derived based on β convergence. This study sets the club convergence of religious diversity as Countries/regions with the same level of RDI at the beginning of the study period to have similar structural characteristics and tend to converge to an approximate local steady-state, while there are spatial differences in the convergence between different types of clubs. Therefore, countries/regions are divided into different continental units on the basis of global absolute convergence, and their convergence differences are tested within them by absolute β convergence.

## Results and analyses

### Spatio-temporal pattern of global RDI and evolutionary characteristics

#### Temporal evolutionary characteristics of global RDI

**Fig 1:**
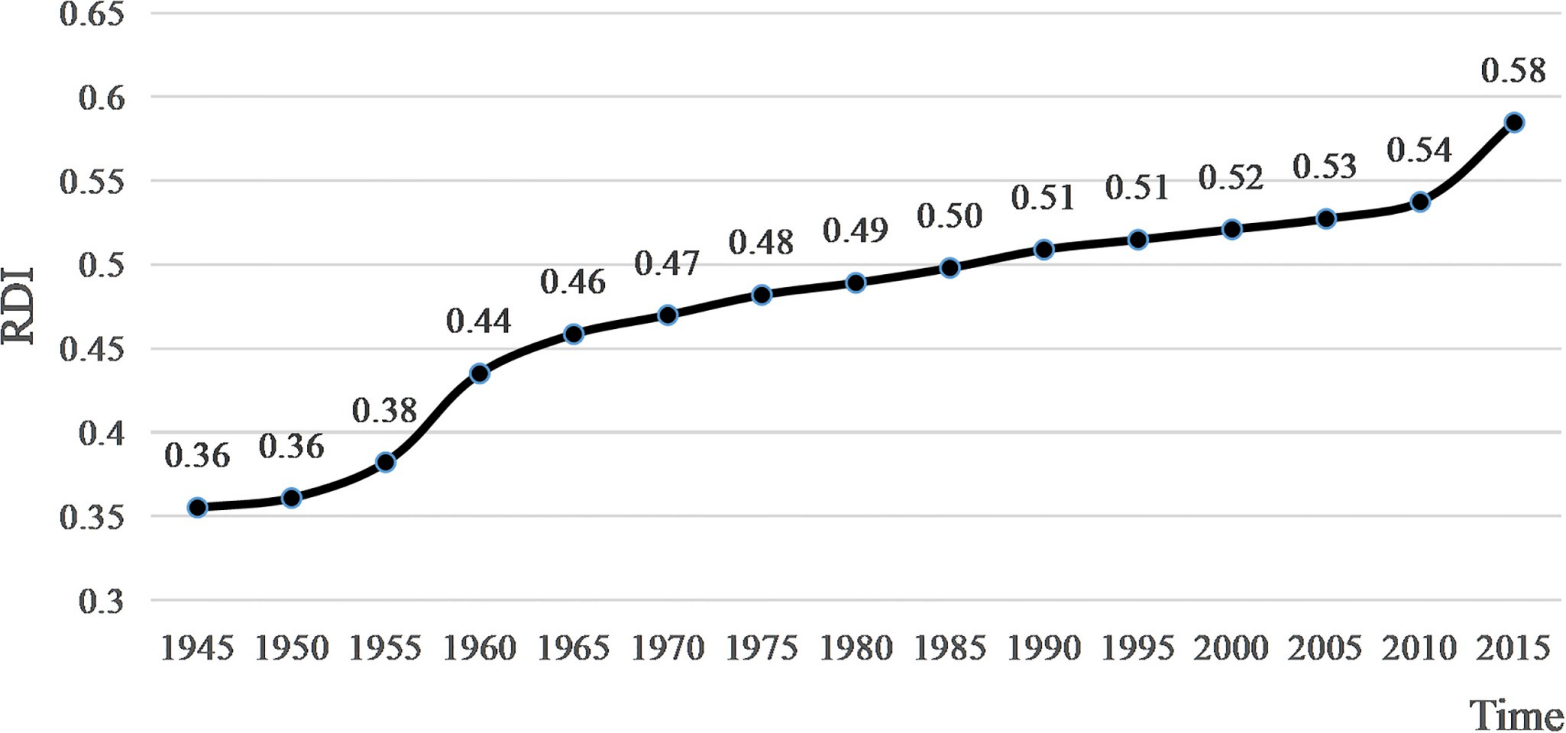
The trend of global RDI after WWII.

Eq (1) was used to calculate the global RDI for each period, and the results are shown in Fig 1. Global RDI has generally shown an upward trend from 1945–2015, increasing from 0.36 in 1945 to 0.58 in 2015, with an increase of 61.11% and an average annual growth rate of about 0.87%. By phase, the 20 years after WWII were the period of the most pronounced increase in global religious diversity, with an average annual growth rate of about 0.50%, much higher than other periods. After that, Global RDI growth began to slow, rising slowly and peaking in 2015.

#### Spatial evolutionary characteristics of global RDI

**Fig 2:**
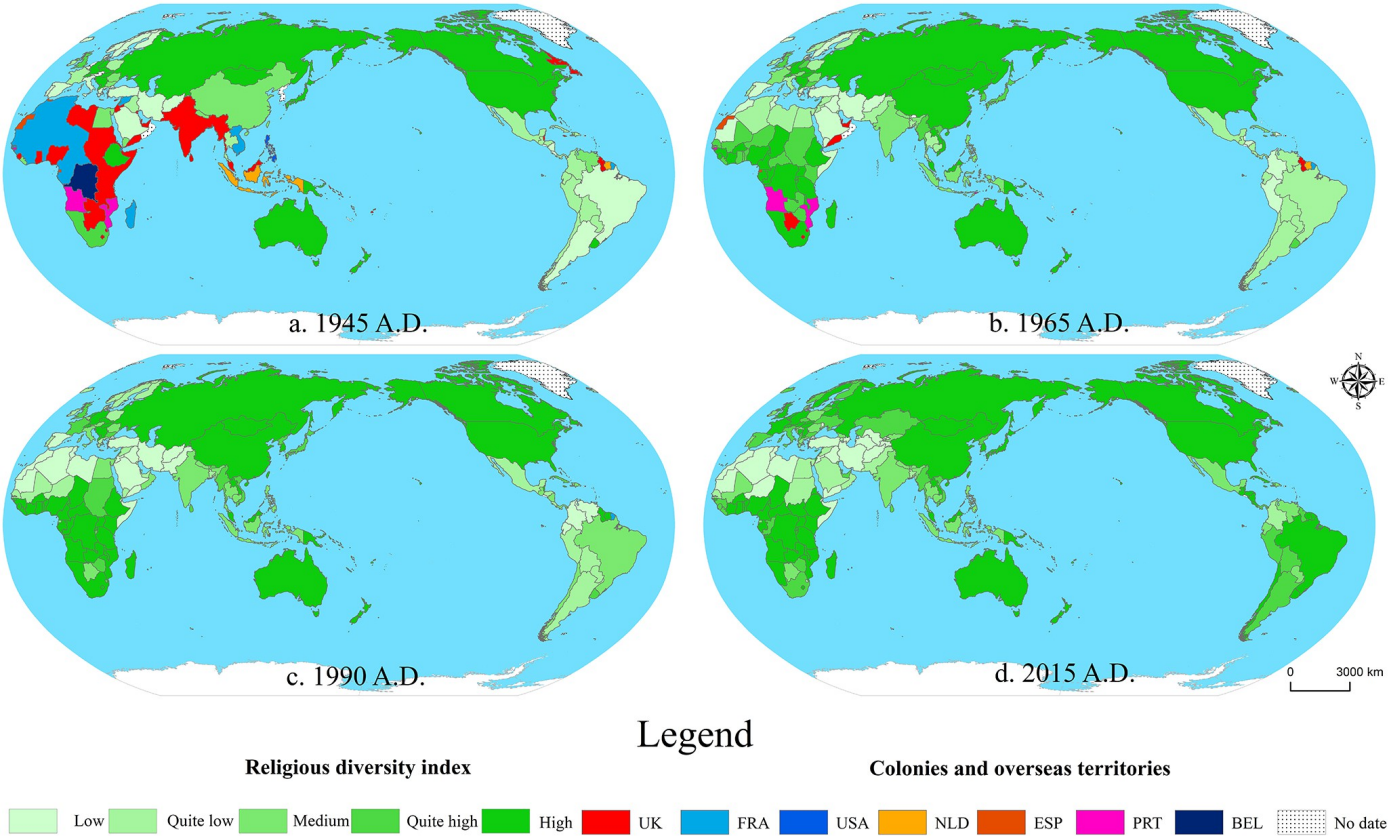


All human social activities, including religious activities, are carried out in a specific space and time. ArcGIS spatial visualization was used to visualize the spatial distribution characteristics and evolution trend of global RDI (Fig 2), and further explore the regional and even national spatial differences. For parallel comparison and to show the magnitude of their growth, the global RDI was divided into five levels: high, quite high, medium, quite low, and low, with 0.2 intervals between them.

As shown in Table 1 and Fig 2, the global level of religious diversity is generally increasing, with most countries/regions achieving at least 1 to 2 levels of improvement during the study period. From 1945–2015, the proportion of high RDI areas in the world increased from 23.08% to 48.19%. The proportion of quite-high value maintained growth of 13.00% despite experiencing some degree of volatility. In contrast, the proportion of low value, which accounted for as much as 29.23% of the study’s base period, decreased rapidly, with only 11.40% remaining as of 2015, a decrease of 17.83%, indicating an overall upward trend in the level of RDI across countries after WWII. Specifically, at the spatial level, the high values of early RDI are concentrated in North America, Eastern Europe, and Oceania, while the low values are distributed in the Middle East and South America. Throughout the study period, RDI in Latin America grew rapidly, by 143.60% over the study period, with an average annual growth rate of 2.05%, due to the dominance of Catholicism before WWII and the rapid spread of Protestant evangelicalism after WWII [42]. Europe, North America and the Caribbean have largely maintained growth curves parallel to global RDI, increasing from 0.47 and 0.33 in 1945 to 0.71 and 0.57 in 2015, respectively. It is important to note that, unlike other regions, only Asia experienced a slight decline in RDI, from 0.51 in 1945 to 0.48 in 2015, a decrease of 3.12%. The main reason is the weakening of indigenous religions in East and Southeast Asian countries due to globalization and the impact of foreign religions after WWII [43].

**Table 1:** Changes in the proportion of countries/regions in different types of RDI after WWII.

Type	1945 proportion	1965 proportion	1990 proportion	2015 proportion	Phase change			
					1945–1965	1965–1990	1990–2015	1945–2015
High	23.08	32.26	41.1	48.19	9.18	8.84	7.09	25.11
Quite high	4.62	16.94	10.43	17.62	12.32	-6.51	7.19	13.00
Medium	13.85	8.87	17.79	13.99	-4.98	8.92	-3.80	0.14
Quite low	29.23	25.00	16.56	8.81	-4.23	-8.44	-7.75	-20.42
Low	29.23	16.94	14.11	11.40	-12.29	-2.83	-2.71	-17.83

#### Country comparisons of global RDI

**Fig 3:**
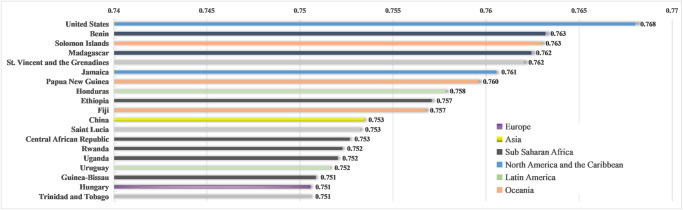
Comparison of RDI of countries regions.

Examining this from a country comparison perspective, we used the bar chart to generate the top 20 countries/regions with the highest RDI in the post-WWII to visually compare the differences in religious diversity on a national scale (Fig 3). As can be seen, the top 20 RDI rankings are, in order, the United States, Benin, Solomon Islands, Madagascar, St. Vincent and the Grenadines, Jamaica, Papua New Guinea, Honduras, Ethiopia, Fiji, China, Saint Lucia, Central African Republic, Rwanda, Uganda, Uruguay, Guinea-Bissau, Hungary, Trinidad and Tobago, ranging from 0.75 to 0.77. Most countries/regions with the highest RDI are located in North America and the Caribbean, and Sub-Saharan Africa, especially in the Pacific and Atlantic islands, and most of them have a history of colonization.

To further explore the deeper information hidden behind the RDI, we quantified the influencing factors of RDI through OLS regression. We selected eight independent variables from three dimensions, including natural conditions, social development status, and historical backgrounds, such as comprehensive national power, GDP per capita in 2015, genetic diversity index, agricultural suitability of land, time of emergence of agriculture and animal husbandry, colonial history duration, missionary effect in the colonial period, and precipitation, to input into the model. The measured model R^2^ was 0.633, and the p-value was 0.000, which was less than the significance level of 0.001 and was consistent with the model hypothesis. The results are shown in Table 2.

**Table 2:** Regression results of influencing factors for RDI.

RDI	Coef.	β	St.Err.	t-value	p-value	Sig	VIF
Missionary effect in the colonial period	0.075	0.410	0.017	4.368	0.000	***	2.312
Colonial history duration	0.000	-0.078	0.000	-0.901	0.370	-	1.954
Comprehensive national power	1.436	0.158	0.580	2.474	0.015	**	1.074
GDP per capita in 2015	0.000	0.355	0.000	4.926	0.000	***	1.359
Genetic diversity index	1.177	0.279	0.381	3.087	0.003	***	2.140
Agricultural suitability of land	0.178	0.182	0.077	2.325	0.022	**	1.606
Time of emergence of agriculture and animal husbandry	-0.081	-0.204	0.031	-2.634	0.010	**	1.573
Precipitation	0.116	0.485	0.022	5.225	0.000	***	2.256
Constant	-0.419	-	0.442	-0.948	0.345	-	-
R-squared	0.633						

Note:

*p<0. 1,

**p<0. 05,

***p<0. 01,

the t-values are in parentheses.

In terms of standardized coefficient (β), missionary effect in the colonial period, precipitation, GDP per capita in 2015, and genetic diversity index were the largest and all had highly significant p-values of 0.410, 0.485, 0.355, and 0.279, respectively. Specifically, the significant positive correlation between precipitation and RDI implies that high RDI areas are globally distributed mainly in tropical and subtropical regions, with decreasing probability of distribution from the equator to the south and north poles. It suggests that improved economic conditions can significantly increase RDI and even social tolerance, which is in line with the assertion by scholars that religiously diverse countries have better economic performance and growth.

That is, even within the same region (especially in relatively poor sub-Saharan Africa and North America and the Caribbean), countries/regions with higher RDI enjoy better economic performance than their neighbors. Regions with high genetic diversity tend to have high RDI, and cultural anthropology theorizes that ethnographic and ethnic differences are the most fundamental group identities of humans, deeply rooted in the nature of society [44]. Different communities often practice different religions to reinforce their identity (e.g., the former northern Sudanese community is predominantly Islamic, while the southern community is largely Christian). The difference between agricultural suitability of land, and the time of emergence of agriculture and animal husbandry in the directionality of the standardized coefficient implies that RDI prefers countries/regions with a shorter history of civilization but superior natural endowments (e.g., the United States).

Also noteworthy is the difference between the two indicators characterizing the colonial background, i.e., the colonial history duration is not significant while the missionary effect in the colonial period is highly significant. It clearly points to the fact that one of the main reasons for the high RDI was the strong cultural output and religious diffusion from the suzerain state during the colonial period. And this religious spread was far stronger and more effective than in other periods. Meanwhile, such colonial imprint has shaped the fundamental underpinnings of today’s world religious landscape, providing evidence for the assertion in quantitative cultural studies of the robust persistence of historical and cultural heritage [45].

#### Analysis of LISA space-time transition in global RDI

The LISA space-time transition was used to represent the transition characteristics and evolution of local spatial association types of global RDI after WWII, and the results are shown in Table 3.

**Table 3:** Local Moran’s I transition probability matrix of RDI after WWII.

Phase	t/t+1	HH	LH	LL	HL	Type	Number	Proportion	SF	SC
1945–1965	HH	0.89	0.07	0.01	0.03	Type 0	285	0.85	0.14	0.86
	LH	0.07	0.55	0.38	0.00	Type I	25	0.07		
	LL	0.01	0.04	0.91	0.05	Type II	22	0.07		
	HL	0.04	0.00	0.15	0.81	Type III	2	0.01		
1965–1990	HH	0.69	0.04	0.01	0.27	Type 0	607	0.84	0.16	0.84
	LH	0.00	0.50	0.50	0.00	Type I	12	0.02		
	LL	0.00	0.08	0.91	0.01	Type II	101	0.14		
	HL	0.03	0.00	0.01	0.95	Type III	3	0.00		
1990–2015	HH	0.67	0.03	0.02	0.28	Type 0	766	0.80	0.17	0.83
	LH	0.13	0.76	0.11	0.00	Type I	79	0.08		
	LL	0.03	0.06	0.87	0.04	Type II	98	0.10		
	HL	0.16	0.00	0.06	0.77	Type III	10	0.01		
1945–2015	HH	0.82	0.04	0.01	0.14	Type 0	1668	0.84	0.15	0.85
	LH	0.08	0.64	0.28	0.00	Type I	86	0.04		
	LL	0.01	0.06	0.89	0.03	Type II	210	0.11		
	HL	0.10	0.00	0.05	0.84	Type III	15	0.01		

During 1945–2015, the probability of Moran’s I scatter of global countries located in the same quadrant (Type 0) was 84.28%, the proportion of Type III transition was extremely low (0.01), without significant data fluctuations or leaping transitions, indicating that the evolution of the spatial structure of global RDI after WWII has considerable stability and path dependence, and the overall fluctuation of religious diversity was relatively small. The total proportion of Type I and Type II does not exceed 20%, which indicates that the overall development of RDI is relatively slow and stable during the study period. Besides, the transition relationships within the transition matrix are relatively balanced, and there are multiple transition possibilities. In terms of the study phase, the spatial dynamics were weakest in the first two decades after WWII and gradually strengthened thereafter, with the proportion of Type 0 decreasing from 85.33% in 1945–1965 to 80.38% in 1990–2015. Correspondingly, the proportion of Type I and Type II also increased from 14.07% to 18.34%, showing the dynamic characteristics of internal transition gradually enhanced. Type III, which represents the synchronous transition between itself and the surrounding regions, is mainly found in the last study phase, showing the overall stability of the study phase as well as the gradual strengthening of the transition rate. The transition probability between transition types shows that there is a proportional relationship between RDI influenced by the neighboring units. It indicates that in addition to its own development, RDI is also influenced by the spillover effects from the neighboring regions.

In terms of the spatio-temporal flow (SF) as well as the spatio-temporal cohesion (SC) of the LISA space-time transition, the spatio-temporal cohesion is much greater than the spatio-temporal flow in each research phase, showing the spatial inertia of the matrix transitions, which is also consistent with the cognition of the relative stability of the development of cultural elements in quantitative social science research [46]. With the development of information and communication technology and the deepening of global integration in the second half of the twentieth century, this spatial stability and path dependence also began to loosen gradually. Furthermore, SC is decreasing while SF is rapidly increasing, and the SF at the end of the study phase is already 22.63% higher than at the beginning of the study phase, which indicates a trend for further improvement of RDI in the future.

Overall, the LISA space-time transition shows a trend of steady development and gradual acceleration of global RDI after WWII. At the same time, this trend is influenced by countries’ development and the spillover effect of the neighboring countries.

### Convergence analysis of global RDI

From the analysis of the LISA space-time transition, it is clear that there is a large spatial difference in the evolution trend of global RDI after WWII. So will the differences between countries/regions and even within regions gradually decrease over time? And is there an identical convergence pattern between them? This section will use convergence to examine the level of global RDI after WWII. The standard deviation evolution trend is first tested by σ convergence to determine whether it converges or not. Then absolute β convergence is used to test whether differences in the level of RDI across countries/regions gradually diminish over time. Finally, the convergence characteristics within its region are examined on this basis with club convergence.

#### σ convergence analysis

**Fig 4:**
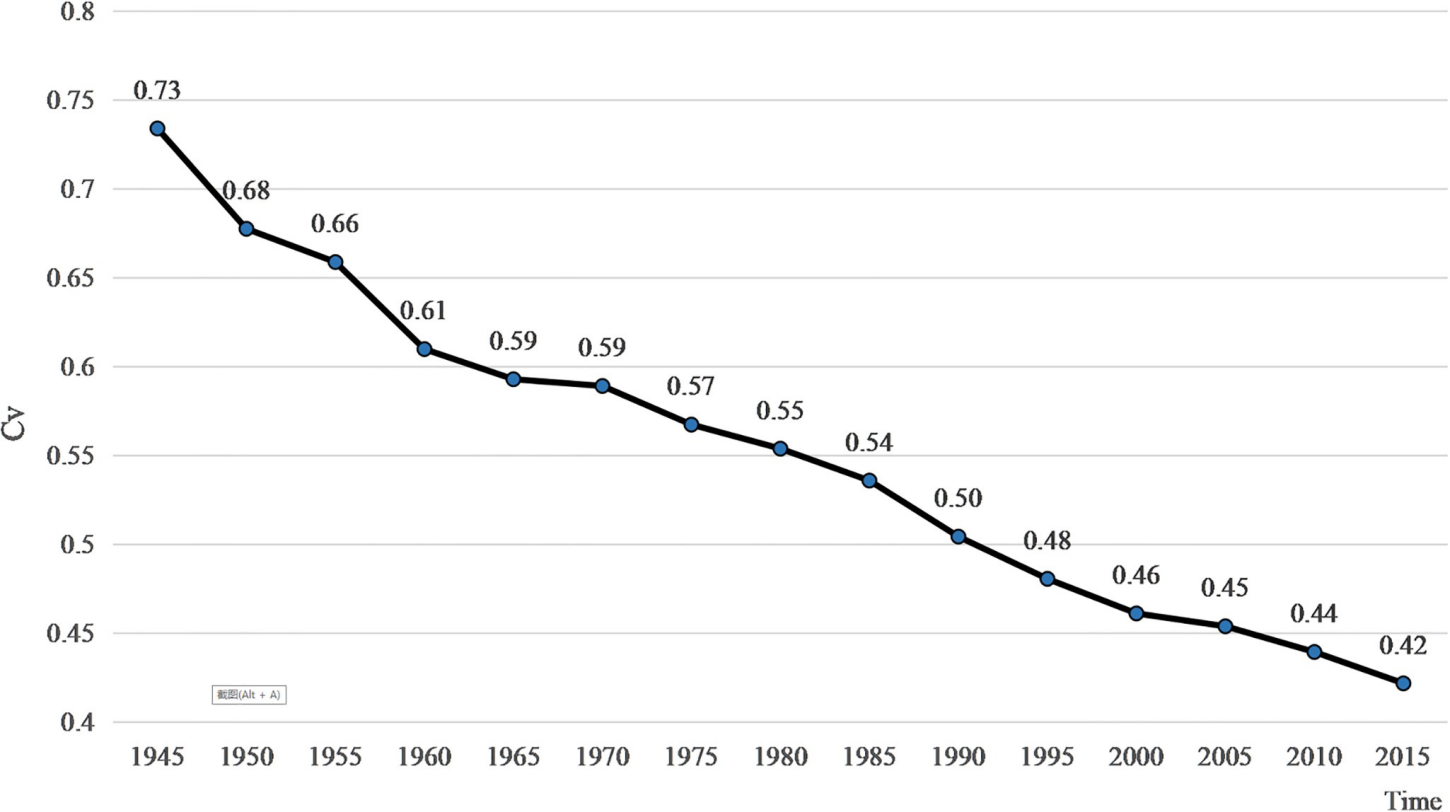
The trend of CV about global RDI after WWII.

Using Stata software, the standard deviation and coefficient of variation (Cv) of the global RDI were calculated for each period from 1945–2015, and the results are shown in Fig 4.

The coefficient of variation of global RDI showed a significant decrease after WWII, from 0.73 in 1945 to 0.42 in 2015, a decrease of 31.23%, proving that there is a significant σ convergence in the level of global RDI within the study period, i.e., the internal differences in the level of global RDI are gradually closing. From the point of view of time, it can be into three stages: 1945–1975, 1975–1980, and 1980–2015, with the decline process showing a rapid, stable, and finally slightly accelerated characteristics. In the first twenty years after WWII, with the arrival of national liberation movements and the “baby boom” in Asian, African, and Latin American countries, the proportion of Islam and other religions increased dramatically, reshaping the pre-war Christian dominance of religion. In addition, the rise of nationalism and the new government’s tendency in supporting traditional polytheistic beliefs curtailed the influence of African Christianity in the first twenty years after WWII. Just as the Reformation in the 16^th^ century stimulated the Catholic Church, it gave rise to newer orders such as the Jesuits who worked to develop unpastoral regions such as the Far East and South America. As a response to this shock, to maintain their position, Christian denominations have made policies such as strengthening religious dialogue and emphasizing religious indigenization to adjust to the changing situation and have reaped results to some extent. Beginning in the 1960s, the proportion of the Christian population rebounded, somewhat curbing the rapid decline in the coefficient of variation. After the 1980s, with the further decline in fertility in Europe and the impact of Islamic revival movements (in particular, Islam developed further during this period in traditionally Islamic countries in Central and Southeast Asia, such as Malaysia and Indonesia), the coefficient of variation began to decline again, at an average annual rate of 0.40% [47].

#### Absolute β convergence analysis

According to Eq (3), we perform the absolute β convergence test on the global RDI for 1945–2015 and by stages. Since this study is mainly based on country/region panel data, the fixed-effects model is chosen for the β convergence model. The results are shown in Table 4.

**Table 4:** Absolute convergence test of RDI after WWII.

Phase	Constant term	Coefficient β	F value	Convergence	Speed θ
1945–1965	0.236***	-0.627***	139.80***	✔	0.097
	(11.817)	(-11.824)			
1965–1990	0.289***	-0.562***	165.22***	✔	0.096
	(13.136)	(-12.854)			
1990–2015	0.453***	-0.930***	412.95***	✔	0.125
	(20.777)	(-20.321)			
1945–2015	0.102***	-0. 239***	138.17***	✔	0.037
	(12.536)	(-11.755)			

Note:

*p<0. 1,

**p<0. 05,

***p<0. 01,

the t-values are in parentheses.

The β coefficients of global RDI levels for 1945–1965, 1965–1990, and 1990–2015 are highly significant and less than 0, indicating significant absolute β convergence of global RDI levels over each study period. The trend shows a decreasing and then increasing speed of convergence in the three periods, from 0.097 in 1945–1965 to 0.096 in 1965–1990, and then back to 0.125 in 1990–2015. Over time, global RDI levels also show absolute beta convergence, i.e., countries/regions with higher levels of RDI have a “spillover effect” on neighboring countries/regions with lower levels, and this diffusion and influence allow the lower countries to catch up the gap. In the post-WWII era, with improved transportation and communication technologies resulting from global infrastructure development, distance, previously the greatest obstacle to efficient communication has been greatly curtailed, and the spread and diffusion of religions have been made easier than ever before [48]. Especially after the 1990s, the rapid development of network technology has enabled the spread of religion beyond natural and administrative boundaries and the original geographical distribution pattern, and this intersection, nesting, and penetration have undoubtedly further increased the level of global RDI to a large extent.

#### Club convergence analysis

Based on absolute β convergence, we grouped global countries/regions into continental units as clubs and tested for club convergence in RDI at the continental scale using a fixed-effects model. The results are shown in Table 5.

**Table 5:** Club convergence test of RDI after WWII.

Regions	Constant term	Coefficient β	F value	Convergence	Speed θ
World	0.102***	-0.204***	138.17***	✔	0.012
	(12.536)	(-11.755)			
Europe	0.022*	-0.013	0.22		—
	(1.750)	(-0.465)			
Asia (except West Asia)	0.241***	-0.482***	90.39***	✔	0.026
	(9.394)	(-9.508)			
West Asia and North Africa	0.101***	-0.483***	80.16***	✔	0.026
	(8.282)	(-8.953)			
Sub-Saharan Africa	0.214***	-0. 363***	104.47***	✔	0.021
	(10.168)	(-10. 221)			
North America and the Caribbean	0.172***	-0. 255***	23.42***	✔	0.015
	(5.187)	(-4.839)			
Latin America	0.042*	-0.061	1.85		—
	(2.658)	(-1.361)			
Oceania	0.515**	-0.731**	6.51**	✔	0.037
	(2.610)	(-2.552)			

Note:

*p<0. 1,

**p<0. 05,

***p<0. 01,

the t-values are in parentheses.

As can be seen from Table 5, except for Europe and Latin America, all regions have negative and highly significant β values, so it can be assumed that there is absolute β convergence in RDI in these regions, and their internal differences will gradually disappear over time and eventually converge to a certain steady-state level. The convergence rates of global as well as continental units are further calculated for the world, Asia (except West Asia), West Asia and North Africa, Sub-Saharan Africa, North America and the Caribbean, and Oceania with 0.012, 0.026, 0.026, 0.021, 0.015, and 0.037, respectively. The fastest convergence was observed in Oceania, while the slowest was observed in North America and the Caribbean, well below the overall global convergence speed. As the continent with the fewest countries, Oceania can be divided into two major parts: Australia and New Zealand, and other Oceanian countries. The fact that internal cultural affinities are much higher than those of other continental units has largely increased the speed of diffusion of cultural elements, including religion, thus rapidly smoothing out the RDI gap within Oceania [49]. In contrast to Oceania, which is more homogeneous, North America and the Caribbean have a similar geopolitical environment to Oceania, but the more complex colonial history and differences in topography make the region internally (e.g., Haiti, Dominica) very different in terms of society, polity, economy, and culture [50]. And the natural barrier of the ocean greatly limits the dissolution of these internal differences, resulting in a slower rate of convergence compared to other regions.

## Conclusion and discussion

Diversity is not only a social phenomenon but also a fundamental condition for guaranteeing religious freedom [41]. Social development has progressively placed difference or diversity as a religious practice in the same important dimension as in the general political life. In the process of human thought, from the existence of religious diversity to the emergence of pluralism that recognizes it more positively, testing the cost and value of freedom. Religious diversity drives religious tolerance to fundamental religious freedom and makes possible freedom of conscience and related human rights in a general sense. At the same time, liberalism’s fundamental value of full recognition of diversity is also a source of continuous enrichment and flourishing of human religious beliefs and practices. This study examines the spatial distribution characteristics, pattern evolution, and convergence of global RDI in the post-WWII at a long time series, global macro scale. The main findings are as follows:

1. At the temporal level, the global RDI increased significantly during the study period, with an increase of 61.11%, and showed a fast and then slow growth trend.
2. At the spatial level, most countries/regions experienced a significant increase in RDI over the study period. The growth of RDI in Latin America is the most obvious, followed by Europe, North America and the Caribbean, and a slight decline in Asia.
3. At the country level, most of the countries/regions with the highest levels of RDI are located in North America and the Caribbean, Sub-Saharan Africa, and most of these countries have a history of being colonized. The OLS regression results showed that RDI was mainly influenced by factors such as the missionary effect in the colonial period, precipitation, GDP per capita, and genetic diversity.
4. The LISA space-time transition shows a certain path dependence in the evolution of the spatial structure of global RDI after WWII, but this trend is gradually weakening. The transition probability between transition types indicates that in addition to countries’ own development, RDI is also influenced by the spillover effects from the neighboring countries.
5. There is significant σ convergence and absolute β-convergence in global RDI levels after World War II, and most of the continental units have club convergence, i.e., the internal differences in RDI levels at the global and continental levels are gradually closing, and there is a spillover effect of higher RDI levels to the surrounding lower regions, and this diffusion or influence allows the lower regions to catch up in the gap of RDI.

Samuel Huntington believes that most conflicts today stem from a clash of civilizations [51]. In addition to the cultural and economic differences that often exist in national and local social structures, religious differences are often an obstacle to social integration and a source of conflict. The rise of religious fundamentalism on a global scale over the past few decades has made the topic of religious diversity one of the keys to analyzing global trends. With the increasing network and informatization, the interaction between religion and globalization is growing, and the opportunities and challenges for global religious development are concurrent. For the world, how to strengthen interfaith dialogue and cooperation, improve global religious governance, form an international collaborative mechanism to resolve religious conflicts, and build a community of faith with interfaith pluralism and harmonious coexistence to promote world peace and development are all becoming important research topics. For this study, there are still some limitations that can be further improved. Firstly, with the refinement and deepening of religious studies from content to research methods, how to model religious populations more scientifically and accurately has become one of the research hotspots [52]. The global data of long time series based on trend interpolation used in this study are more suitable to be applied for analysis at the macroscopic level, while there is a lack of expressiveness at the microscopic scale. Secondly, we did not further analyze the future trends of RDI due to the length limitation of the paper. Future simulations that are more realistic considering many objective constraints should be introduced into the next research work.
